# A Hypothesized Mechanism for Chronic Pancreatitis Caused by the N34S Mutation of Serine Protease Inhibitor Kazal-Type 1 Based on Conformational Studies

**DOI:** 10.2147/JIR.S304787

**Published:** 2021-05-21

**Authors:** Martin Kulke, Felix Nagel, Lukas Schulig, Norman Geist, Marcel Gabor, Julia Mayerle, Markus M Lerch, Andreas Link, Mihaela Delcea

**Affiliations:** 1 Institute of Biochemistry, University of Greifswald, Greifswald, Germany; 2 Institute of Pharmacy, University of Greifswald, Greifswald, Germany; 3 Department of Medicine II, Ludwig-Maximilian University of Munich, Munich, Germany; 4 Department of Medicine a, University Medicine Greifswald, Greifswald, Germany

**Keywords:** trypsin, molecular dynamics simulations, replica exchange, transition path sampling, umbrella sampling

## Abstract

**Purpose:**

Although strongly related, the pathophysiological effect of the N34S mutation in the serine protease inhibitor Kazal type 1 (SPINK1) in chronic pancreatitis is still unknown. In this study, we investigate the conformational space of the human cationic trypsin-serine protease inhibitor complex.

**Methods:**

Simulations with molecular dynamics, replica exchange, and transition pathway methods are used.

**Results:**

Two main binding states of the inhibitor to the complex were found, which explicitly relate the influence of the mutation site to conformational changes in the active site of trypsin.

**Conclusion:**

Based on our result, a hypothesis is formulated that explains the development of chronic pancreatitis through accelerated digestion of the mutant by trypsin.

## Introduction

Pancreatitis is an inflammatory disorder of the pancreas. While acute and chronic pancreatitis were previously viewed as separated diseases, today they are regarded as a continuum, with nearly 30% of the patients exhibiting overlapping phenotypes that manifest as recurrent pancreatitis.^1^ Chronic pancreatitis (CP) develops from recurrent episodes of acute pancreatitis that lead to fibrosis, exocrine pancreatic insufficiency and diabetes. While initial triggers for pancreatitis are diverse, almost all of them result in premature activation of trypsin and subsequently other proteases in pancreatic acinar cells. Therefore, pancreatitis is considered to be an autodigestive disorder caused by trypsin auto-activation, which is also supported by numerous mutations in the *PRSS1* gene coding for human cationic trypsin (TRY1).^2^,^3^ The serine protease inhibitor Kazal-type 1 (SPINK1, also known as PSTI or TATI) represents the first line of defense against the trypsin auto-activation cascade by potently binding and inhibiting active trypsin. The inhibitor has a size of 6.2 kDa and is co-located with trypsinogen and other zymogens in storage organelles called zymogen granules of pancreatic acinar cells. The c.101A>G point mutation is the most common variant of the *SPINK1* gene, which results in a p.N34S amino acid substitution and represents one of the most clinically relevant risk factors for chronic pancreatitis with almost 10% of the patients carrying the mutation, compared to 1% of the healthy population.^4–6^ Although the p.N34S mutation was identified over two decades ago and was subjected to many additional studies, the mechanism of action of this variant remains enigmatic to this day.^4^,^5^,^7^,^8^ Hypotheses regarding the disease relevant function of this variant ranged from reduced inhibitory activity, misfolding, reduced expression or secretion to reduced proteolytic stability, structural alterations and secondary trigger mechanisms. While most of the aforementioned hypotheses have now been refuted, very little is known about the SPINK1 structure.^9–16^

Patient studies revealed that the p.N34S mutation alone is not sufficient to develop chronic pancreatitis, but must be combined with additional risk factors.^17–19^ It can therefore be assumed that the p.N34S mutation does not lead to a complete destruction of functional SPINK1, but rather reduces either the kinetic properties or the number of correctly folded SPINK1 molecules. In an earlier study, we showed differing secondary structures of SPINK1 wild type (WT) and the N34S mutant by circular dichroism (CD) spectroscopy as well as different secondary structures at lower pH values.^20^ However, these minor changes could not be linked to different inhibitory activities of the two variants. The location of differing regions within the proteins could also not be precisely determined, which makes it difficult to assess the relevance of the relatively small changes in the secondary structure and their possible influence on the tertiary structure of the inhibitor. It also remains unclear how the structures of SPINK1 and its N34S mutant behave in complex with its natural target trypsin (TRY1). In a recent study by Sun et al, the SPINK1-TRY1 complex structure was examined by docking and molecular dynamics simulations and no significant difference in secondary structure content was reported, but the loop region around the mutation site was more flexible compared to the wild type.^21^

In addition, the complex association and dissociation rates of mutant and WT were examined and found to be the same within the margin of error.^11^,^20^ They argued that although these constants are experimentally indistinguishable, their theoretical results support a slight deviation of these rates, which leads to a lower inhibition potential of the p.N34S-mutated SPINK1.^21^

**Figure 1:**
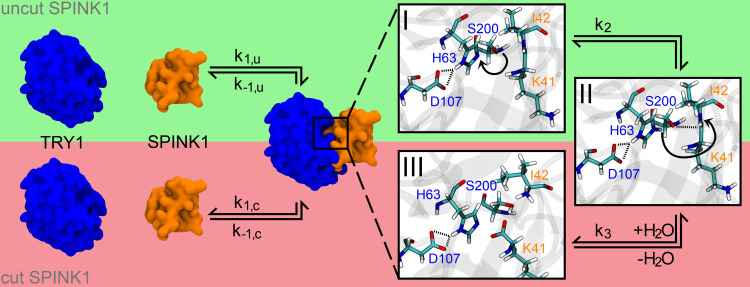
Complex formation for SPINK1 (orange) to TRY1 (blue) including the deactivation pathway of SPINK1 by cutting the K41-I42 peptide bond. The rate constants for the complexation/decomplexation of cut and uncut SPINK1 are indicated with *k*_1,u_/*k*_−1,u_ and *k*_1,c_/*k*_−1,c_, respectively. The zooms on the right partially show the cutting mechanism of SPINK1, which is catalysed by the catalytic triad S200-H63-D107 of TRY1. Starting from state I, the hydroxy hydrogen atom is translated to the far ring nitrogen position of H63 to state II (rate constant *k*_2_). Afterwards, the deprotonated hydroxy oxygen atom of S200 coordinates to the carboxamide carbon atom of K41, while the carboxamide nitrogen atom (I42) attacks the previously translated hydrogen of H63 and the peptide bond is cleaved (rate constant *k*_3_). The system is finally recovered by the addition of water to state III (not shown here).

In the present study, we used molecular dynamic simulations (MDS) to elucidate structural and kinetic features of the SPINK1-TRY1 complex and differences due to the p.N34S amino acid substitution in SPINK1. In particular, the secondary structure content in the complex and solution structure of SPINK1 for WT and the p.N34S mutant is examined. For the first time, the inactivation mechanism of SPINK1 through hydrolysis of K41-I42 is investigated and possible differences between WT and mutant are clarified (Figure 1). Based on these results, a hypothesis is formulated that explains a reduction in active p.N34S-mutated SPINK1 compared to WT.

## Materials and Methods

### Model for the SPINK1-TRY1 Complex

To obtain the SPINK1-TRY1 complex, the crystal structure of human cationic trypsin (PDB code: 1TRN)^22^ was superposed onto the structure of bovine chymotrypsinogen A in complex with SPINK1 (PDB code: 1CGI).^23^ The mutation sites K41Y, I42E and D44R in SPINK1, which were introduced to stabilize the complex structure during crystallization, were mutated back to the WT amino acids. In addition, Y154 was phosphorylated by TRY1 according to 1TRN^22^ and a structural calcium ion was added to the coordination site E75-N77-V80-E85. The resulting structure was solvated in explicit water, briefly minimized and equilibrated. The protonation states of the amino acids were then calculated using the H++ server assuming a pH of 7.^24^ In addition, a physiological sodium chloride concentration of 0.15 M was added while the total charge in the simulation cell was neutralized. Finally, after adjusting the temperature and pressure in an NVT followed by an NPT ensemble, the complex was equilibrated for 21 ns in an NPT ensemble.

### Molecular Dynamics Simulations

The SPINK1-TRY1 system was theoretically investigated with molecular dynamic simulations using the AMBER99SB-ildn force field,^25^ which was specially developed for the representation of proteins. The equation of motion was integrated with a Verlet integrator every 2 fs using the GROMACS-5.1 software package.^26^ The non-standard amino acid phosphotyrosine was represented with the FFPTM force field,^27^ while water is defined by the TIP3P model.^28^ The system is simulated with periodic boundary conditions in all spatial directions and the center of mass movement is removed every 200 fs. The temperature is set to 310 K for protein and non-protein separately by a modified V-rescale thermostat^29^ with a coupling constant of 100 fs in order to prevent overcooling of the protein. The pressure is controlled by a Berendsen barostat^30^ to 1 bar with a coupling constant of 1 ps and isotropically pushing against the system by assuming a compressibility of 4.5e^−5^ bar^−1^. During the simulation, all bonds are constrained to their optimal length using the LINCS algorithm.^31^ Non-bonded interactions are considered with 12-6-Lennard-Jones and Coulomb functions up to a distance of 1 nm and the fast smooth Particle-Mesh-Ewald method with a 1 nm cutoff in direct space and 0.12 nm grid spacing in reciprocal space.

Starting from the equilibrated TRY1-SPINK1 system, three independent simulations were carried out for different simulation setups: 1) TRY1 in complex with WT SPINK1, 2) TRY1 in complex with p.N34S mutated SPINK1, 3) WT SPINK1 in solution, 4) p.N34S mutant SPINK1 in solution, 5) TRY1 in complex with WT SPINK1, but the S200 of TRY1 was deprotonated and the hydrogen was moved to the nitrogen of H63 to simulate an activated catalytic triad (Figure 1, state II) and 6) as 5) but with p.N34S mutated SPINK1. All systems were again equilibrated before collecting data for at least 50 ns. The partial charges for deprotonated serine were determined with the standard Amber protocol using Gaussian 2003.^32^

All simulated protein trajectories are visualized, atom-to-atom distances are analyzed and porcupine diagrams are carried out with VMD 1.9.2.^33^ The reaction coordinate was determined by aligning the protein backbone of 14 frames along the trajectory of the TRY1-N34S SPINK1 simulation, which included the flip of N37. After performing a coordinate principal component analysis of these frames using only the backbone of amino acids K102, K178, W216 of TRY1 and N34S, E35, N37, R65 of SPINK1, the first principal component is the reaction coordinate. The secondary structure content was estimated using the CPPTraj program.^34^

### Replica Exchange Simulations

Replica exchange simulations based on the TIGER2hs^35^ method were carried out for the equilibrated simulation setups 5) and 6). The extended sampling algorithm is implemented in the NAMD 2.13^36^ engine and CUDA acceleration. GROMACS topologies and coordinate files were transformed into the Amber format using ParmED,^37^ while additionally applying the hydrogen mass partitioning scheme (HMR)^38^ to the solute. The time step was increased to 4 fs, whereby the RATTLE^39^ algorithm fixed the lengths of all bonds to hydrogen atoms. Short-range interactions based on Lennard-Jones and Coulomb potentials used a 0.9 nm limit and 0.1 nm switching function. Long-distanceelectrostatics were described by particle mesh Ewald (PME) with a lattice spacing of 0.1 nm. The temperature was adjusted by a Langevin thermostat^40^ with a damping coefficient of 1 ps^−1^, while a Langevin piston barostat^41^ applied pressure control to 1 bar at 100 ps intervals with a 200 ps decay time constant. A temperature range of 300–370 K was spanned over 32 replicas in a pseudo-NVT ensemble.^42^ A TIGER2hs sampling cycle consisted of 16 ps rapid heating and sampling followed by 4 ps rapid quenching and cooling. Afterwards, the hybrid solvent energy was calculated. The amount of water molecules in the first two water layers was determined by analyzing the radial distribution function along the surface of the protein after a short MD simulation at 310 K. 1080 explicit water molecules were considered for the hybrid solvent exchange phase.

Implicit solvent energies are evaluated by the Gb_obcII_^43^ model using OpenMM 7.4^44^ and periodic boundary conditions. Non-bonded cutoffs were set to half the cell size. Exchange attempts were carried out between the current baseline and another randomly selected replica after the cooling phase according to the TIGER2 scheme.^45^ In each cycle, 32 exchange attempts were performed, while immediately swapping the temperatures of replicas after a successful attempt. The conformations from each successful exchange to the baseline were written to the trajectory. Before the next simulation cycle, all non-baseline replicas were assigned new temperatures based on their respective potential energy order.

### Transition Path Sampling

Starting from the equilibrated system setups of 5) and 6), umbrella sampling simulations^46^ along the reaction coordinate were performed using OpenMM 7.4.^44^ Thereby, the reaction coordinate was separated in evenly distributed values ranging from 1 to −1 in 0.05 steps with positive and negative values relating to state A/B and C/D, respectively. To obtain structures on the respective reaction coordinate bins, referred to as umbrellas, a harmonic force was applied in the reaction coordinate space with a force constant of 400 kJ mol^−1^ nm^−1^, while constraining the structure in place through position restraints on C_α_ atoms in β-sheets and the geometric center defined by the amino acids K102, K178, W216 of TRY1 and N34S, E35, N37, R65 of SPINK1. Each umbrella was subjected to a 600 ps equilibration in a NPT ensemble with the temperature and pressure set to 310 K by a Langevin thermostat and 1 bar by a Monte Carlo barostat, respectively. Afterwards, each umbrella was sampled for 1 ns, while reducing the force constant for the reaction coordinate restrain in two separate runs to 5 and 10 kJ mol^−1^ nm^−1^. The result was analyzed with the weighted histogram analysis method (WHAM).^47^

## Results

### Conformations in the Proximity of the Mutation Site

**Figure 2:**
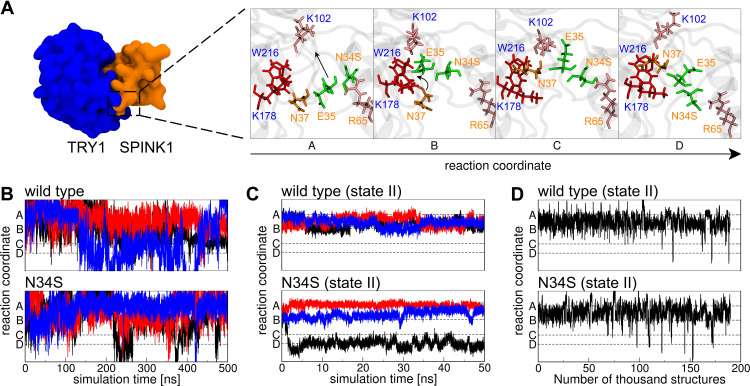
(**A**) The reaction coordinate as observed in one TRY1-N34S SPINK1 molecular dynamics simulation (cf. (**C**) black curve). Blue and orange labels indicate amino acids from TRY1 and SPINK1, respectively. The amino acids in stick representation are colored by their supposed functions, red binding pocket, orange key, pink steric barrier and green amino acids that have to pass the barrier. (**B**) Conformational state in relation to the reaction coordinate for SPINK1 in solution for WT and N34S mutant. The colors black, blue and red relate to three independent simulations. (**C**) Same as (**B**), but for SPINK1 in complex with TRY1 in state II (cf. Figure 1). (**D**) Conformational state in relation to the reaction coordinate of structures for the TRY1-SPINK1 complex in state II (cf. Figure 1) observed during a replica exchange simulation.

The secondary structure content of SPINK1 in complex with TRY1 and free in solution was analyzed. In contrast to the circular dichroism data presented in Buchholz et al,^20^ the secondary structure content of SPINK1 does not differ significantly between the N34S mutant and WT (Table 1). In the complexed state, there are also no differences in the secondary structure content, but we found that the loop region spanned by amino acids 34–38 of SPINK1 has distinct conformations due to the steric hindrance caused by the binding. From these different states, a reaction coordinate is defined that covers the structures A–D (Figure 2A). Typically, the major binding states are A and B, which can also be found in the crystal structure.^22^ However, in 1 of 12 simulations of the complex, a change to the states C and D was observed. The structure was stable and did not fold back to A/B (Figure 2C, black N34S curve). As this observation is either a rare event or an artifact of the simulation, replica exchange molecular dynamics simulations (REMD) were performed to better understand the state distribution of the TRY1-SPINK1 complex. Several states were found in C and D in the resulting structural ensemble, supporting the argument that this observation is rare (Figure 2D). Unfortunately, the REMD simulation did not fully converge and the computational resources required to perform a converged simulation were beyond reasonable considerations. Therefore, these data can only be discussed qualitatively and the correct state populations are not accessible via REMD.

**Table 1:** Secondary Structure Content Averaged for All SPINK1 Amino Acids for the TRY1-SPINK1 Complex and Unbound SPINK1 (System Setups 1)–4))

	TRY1-SPINK1		SPINK1 in Solution	
	Wild Type	N34S	Wild Type	N34S
Helix	19%	19%	19%	20%
Sheet	18%	19%	18%	17%
Turn/bend	24%	23%	23%	23%
Coil	39%	39%	40%	40%

### Conformational Changes of the Complex Based on the Reaction Coordinate State

**Figure 3:**
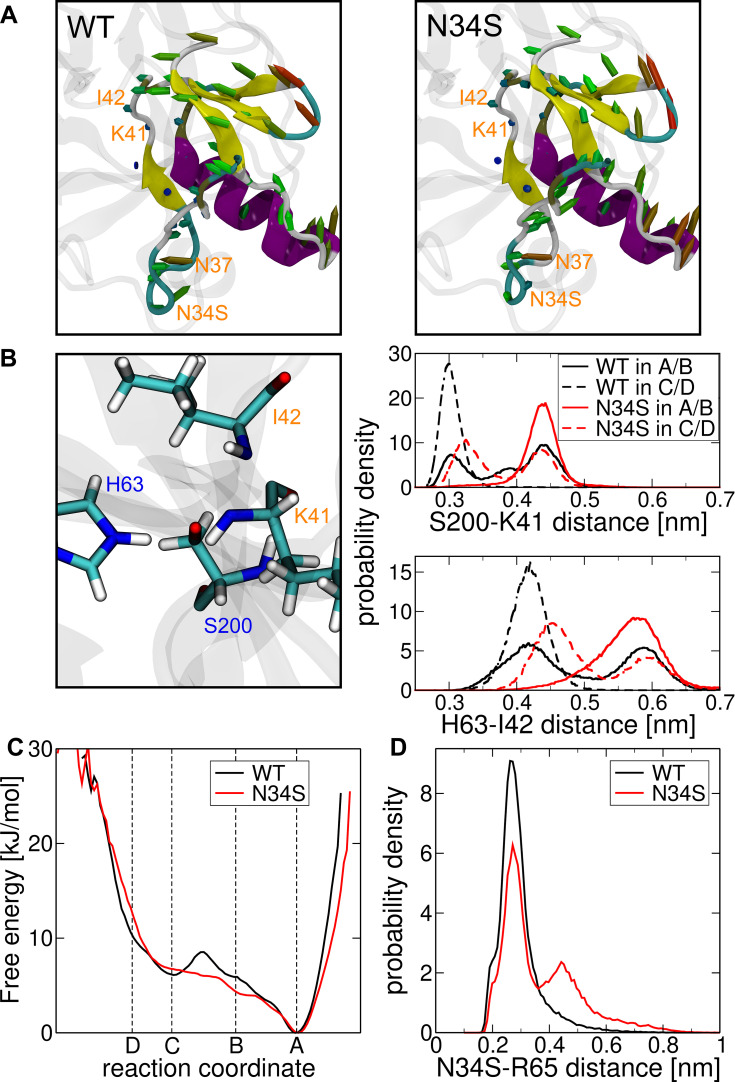
(**A**) Porcupine plots that show the structural difference when state II A/B is transitioned to state II C/D for the mutant right and WT left (cf. Figures 1 and 2A). The colors indicate the secondary structure elements for helix purple, sheet yellow, turns cyan and coil white. The marked arrows indicate the transposition of the C_α_ atoms with the color gradient blue-green-red for small-medium-large deviations, respectively. (**B**) Distance distributions in the active center between the hydroxy oxygen atom of S200 and the carboxamide carbon atom of K41 (top), and between the far ring hydrogen of H63 and carboxamide nitrogen atom of I42 (bottom). The distributions are measured from respective REMD simulations in state II A/B and state II C/D of the mutant (N34S) and wild type (WT). (**C**) Free energy in kJ/mol along the reaction coordinate determined by transition path sampling of the wild-type solid and mutant dashed. (**D**) Histogram of the minimal distance between the amino acids N34S and R65 for wild-type black and mutant red.

To identify the conformational changes associated with the change in the reaction coordinate (Figure 2A), the last 50,000 conformations from REMD simulations of the TRY1-SPINK1 complex were aligned with the backbone of TRY1 and averaged. Simulations were carried out for systems of the N34S mutant and the wild type in state A/B and C/D, respectively. The visualization of the porcupine plots shows that in the transition from state A/B to state C/D, the overall structure of SPINK1 in the binding pocket for both SPINK1 forms rotates counterclockwise with respect to the SPINK1-TRY1 viewing axis (Figure 3A). This leads to interesting aspects of the relevant atom-atom distances for the degradation. The distance between the hydroxy hydrogen atom of S200 and the far ring nitrogen of H63 for state I and II in A/B and C/D is always about 0.2–0.25 nm, which indicates that the reaction from state I to state II is fast and reversible. In state II, however, the distances between the hydrogen of the distant ring of H63 and the carboxamide nitrogen atom of I42 and between the hydroxy oxygen atom of S200 and the carbon atom of the peptide bond of K41 show great differences. They are both significantly reduced by going from state II A/B to C/D for WT and mutant (Figure 3B). In addition, these distances are generally smaller in the WT form.

## Discussion

### The Reaction Coordinate Includes a Major Energy Barrier

State A is stabilized by the polar interaction between N34S and R65 and is characterized by N37 and E35 being in a downward position with respect to amino acids W216 and K178 of TRY1 (Figure 2A). In state B, the N34S-R65 interaction breaks, causing E35 to move upwards. This allows N37 to reach a binding pocket that is spanned by K178 and W216 in state C. This conformation is stabilized by an interaction formed between E35 and K102. Finally, in state D, E35 moves back down to lock N37 in the binding pocket. In our opinion, the highest energy barrier for the transition between A/B and C/D is given by the rigidity of the system, and amino acids K102 and K65 act as gatekeepers preventing E35 from moving straight up. However, E35 has to move away for N37 to reach the upper binding pocket as the bulky amino acids K178 and W216 prevent the movement through them.

Sun et al also examined the loop region around the N34S mutation site in molecular dynamic simulations involving the chymotrypsinogen A-SPINK1 complex.^21^ They observed greater flexibility in the WT compared to the N34S mutant of this loop and argued that the mutation rigidifies the loop. We believe that their data is explained by the rare event described by the reaction coordinate. Although they performed more extensive molecular dynamics simulations on the complex with a total simulation time of 5 μs, the event may only be observed a few times, resulting in non-converged ensembles for their WT and N34S mutant simulations. If the event occurs more frequently in the WT, the flexibility of the loop is higher because the transitions along the reaction coordinate result in a significant change in the loop dihedral angles (Figure 2A). This suggests that the loop in the mutant is more rigid.

### Hypothesis for Differences in the Inhibition Behavior of the N34S Mutant

In formulating a hypothesis as to why the N34S mutant is a risk factor for chronic pancreatitis, the following assumptions are made. First, it is assumed that the binding of mutant and WT SPINK1 to TRY1 occurs mainly in state A or B (Figure 2A), as found in the crystal structure of bovine chymotrypsinogen A in complex with SPINK1 (PDB code: 1CGI). In this context, it should be noted that in solution the mutant loop region of SPINK1 frequently crosses all states A–D (Figure 2B). This does not mean that the binding occurs in a random state as there is most likely a preference, but our data does not support any statements about the exact probabilities. Secondly, phosphorylated TYR-154 as found in the crystal structure (PDB code: 1TRN)^22^ is used in the model instead of sulfated^48^ and it is assumed that both post-translational modifications exhibit the same effects in regard to the analyses performed in this study. While the effect of the sulfation is not clear in human cationic trypsin and phosphorylated and sulfated TYR are chemically similar, sulfation instead of phosphorylation can lead to slight differences in the binding conformations.^49^ The third assumption is that the difference between mutant and WT in terms of developing chronic pancreatitis is mainly kinetic in nature. This is supported by the fact that no conclusive conformational differences were found in the present or previous studies. The mutation also does not result in a misfolding or a complete absence of SPINK1, as this mutation alone is not enough to develop chronic pancreatitis and the mutation is also present in the healthy population. Contrary, there is experimental evidence that the N34S mutant may be expressed significantly less than the wild type, which would also explain the development of chronic pancreatitis.^50^

In previous studies, kinetic inhibition and surface plasmon resonance spectroscopy experiments revealed that the association and dissociation constants of the SPINK1-TRY1 complex were not significantly different between WT and N34S.^11^,^20^ A recent study to the digestion of SPINK1 mutant and wild type by mesotrypsin showed also no difference.^51^ To the best of our knowledge, there is no current study that has investigated the kinetics of peptide hydrolysis for this system. Hence, the rate constants k_2_, k_3_, k_1,c_ and k_−1,c_ could show differences between mutant and WT (Figure 1). A second consideration is that the rate constant in question should be the slowest of these constants to have the necessary effect. Since digestion appears to be the slowest process and the transition from state I to state II is fast and reversible, we focus on the rate constant k_3_. At this point, it can be assumed that digested SPINK1 has a significantly lower inhibitory potential than active SPINK1, since the inhibition strength of SPINK1 in enzyme tests diminishes measurably over time.^52^

Figure 3B shows that the average distances that are important for the digestion of SPINK1 in the active center are significantly smaller in state II C/D compared to state II-A/B. This means that SPINK1 is digested faster in state II C/D and that the transition from state A/B to C/D is a necessary activation step for digestion. While the populations of state A–D are similar for WT and mutant, the energy barrier between the transition from A/B to C/D is significantly higher for the WT (Figure 3C). We believe that this is related to the N34S-R65 interaction in SPINK1 (Figure 2A). Since the side chain of serine at the mutation site is shorter than that of asparagine, the average minimum distance increases and thus, the strength of the interaction is weakened (Figure 3D). This makes it easier for amino acid E35 to move upwards (Figure 2A–C) and lowers the barriers from A to B and especially from B to C. We propose that this step is the rate-limiting step in the digestive process and therefore the mutant is digested faster, because of the reduced energy barrier between states A/B and C/D.

This statement is of course debatable, as our data does not support conclusive explanations for the rate constant differences between the transition from A/B to C/D and the digestion in state II C/D. In addition, it is unclear whether the digestion rate for the WT in state II A/B is slower than for the mutant in state II C/D (Figure 3B). In this regard, we assume that the energy barriers in Figure 3C are significantly higher for both WT and mutant, supporting the argument that this step determines the rate. This is due to the transition path sampling method used for this data. It depends on the force constant applied and the step size, as well as the atomic position constraint. Based on the restraint methods used, the atomic positions could still perform minor movements, which can lead to smaller measured forces, especially in high-energy states. This leads to a general reduction of the measured energy barriers between states. However, we expect this error to affect WT and mutant in the same way.

## Conclusion

In the present study, the conformations of the TRY1-SPINK1 complex were examined in detail using molecular dynamic simulations. Two binding populations A/B and C/D were identified, separated by a major energy barrier, which is significantly lower in the case of the N34S SPINK1 mutant. It has been argued that this leads to a weaker TRY1 inhibition strength of the mutant, as it enables a faster digestion of SPINK1 by TRY1 compared to the WT. The authors want to clarify that the proposed mechanism is hypothetical and needs to be further validated in future experimental and theoretical studies. Nevertheless, it might be an explanation for incomplete penetrance of the disease phenotype in patients with chronic pancreatitis and a N34S mutation. Since the hypothesis predicts that the mutant will be digested more quickly, a similar enzyme assay as in^51^ for N34S and WT SPINK1 with TRY1 can refute or support the proposed mechanism. The second prediction is that amino acids K102 of TRY1 and E35, N37 and R65 of SPINK1 play an important role in the digestion of SPINK1. The N37S and R65Q mutants showed normal binding behavior to TRY1,^51^ but it will be interesting to investigate these mutants in the context of digestion. Lastly, in this study, it is assumed that the state of binding of N34S mutant SPINK1 to TRY1 is similar to the state of binding of WT SPINK1 to bovine chymotrypsinogen A. A crystal structure of the N34S SPINK1-TRY1 complex will clarify this assumption.

Finally, a better understanding of the digestive mechanism of SPINK1 caused by TRY1 can help to develop a more robust SPINK1 mutant that is hardly digested and can act as a drug for chronic pancreatitis caused by SPINK1.
